# Sex Disparities in Receipt of Bystander Interventions for Students Who Experienced Cardiac Arrest in Japan

**DOI:** 10.1001/jamanetworkopen.2019.5111

**Published:** 2019-05-31

**Authors:** Satoshi Matsui, Tetsuhisa Kitamura, Kosuke Kiyohara, Junya Sado, Mamoru Ayusawa, Masahiko Nitta, Taku Iwami, Ken Nakata, Yuri Kitamura, Tomotaka Sobue

**Affiliations:** 1 Department of Emergency Medicine, Hyogo Prefectural Kobe Children’s Hospital, Hyogo, Japan; 2 Division of Environmental Medicine and Population Sciences, Department of Social and Environmental Medicine, Graduate School of Medicine, Osaka University, Osaka, Japan; 3 Department of Food Science, Otsuma Women’s University, Tokyo, Japan; 4 Medicine for Sports and Performing Arts, Department of Health and Sport Sciences, Graduate School of Medicine, Osaka University, Osaka, Japan; 5 Department of Pediatrics, Nihon University School of Medicine, Tokyo, Japan; 6 Department of Emergency Medicine, Osaka Medical College, Osaka, Japan; 7 Department of Pediatrics, Osaka Medical College, Osaka, Japan; 8 Kyoto University Health Service, Kyoto, Japan

## Abstract

**Importance:**

Bystander interventions are a factor for improving survival of out-of-hospital cardiac arrest (OHCA), but it is hypothesized that girls and women experiencing OHCA may be less likely to receive bystander interventions than boys and men.

**Objective:**

To investigate sex disparities in receiving public-access automated external defibrillator (AED) pad application and bystander-initiated cardiopulmonary resuscitation (CPR) among students who experienced OHCA in school settings.

**Design, Setting, and Participants:**

This nationwide cohort study used the Stop and Prevent Cardiac Arrest, Injury, and Trauma in Schools (SPIRITS) database to link databases from 2 nationally representative registries—the Injury and Accident Mutual Aid Benefit System of the Japan Sport Council and the All-Japan Utstein Registry of the Fire and Disaster Management Agency. Students from elementary schools (ages 6-12 years), junior high schools (ages 12-15 years), high schools (ages 15-21 years), and technical colleges (ages 15-21 years) who experienced nontraumatic OHCA involving attempted resuscitation by emergency medical service personnel or bystanders in school settings from April 1, 2008, to December 31, 2015, were included. Data analysis was performed from January 5, 2019, to April 11, 2019.

**Exposures:**

Sex and school level.

**Main Outcomes and Measures:**

Application of public-access AED pads or initiation of CPR by a bystander.

**Results:**

A total of 232 students who experienced OHCA with nontraumatic causes in school settings (mean [SD] age, 14.5 [2.9] years; 175 [75.4%] male) were included. In multivariable analysis of the full cohort of students who experienced OHCA, female sex was associated with significantly lower odds of receiving public-access AED pad application compared with male sex (36 of 57 female students [63.2%] received AED pad application vs 141 of 175 male students [80.6%]; adjusted odds ratio [OR], 0.44; 95% CI, 0.20-0.97; *P* = .04). In the subgroup analysis of students who experienced OHCA in high schools or technical schools, female sex was associated with significantly lower odds of receiving public-access AED pad application compared with male sex (10 of 18 female students [55.6%] vs 84 of 101 male students [83.2%]; adjusted OR, 0.26; 95% CI, 0.08-0.87; *P* = .03). Among the full cohort, 48 of 57 female students (84.2%) and 151 of 175 male students (86.3%) received CPR from bystanders (adjusted OR, 0.81; 95% CI, 0.30-2.22), and there were no significant differences in receiving bystander-initiated CPR between sexes, irrespective of school level.

**Conclusions and Relevance:**

Among students who experienced OHCA in schools in Japan, female sex was associated with lower odds of receiving public-access AED pad application compared with male sex.

## Key Points

**Question:**

Are there any sex disparities in receiving bystander interventions among students who experience out-of-hospital cardiac arrest (OHCA) in school settings?

**Findings:**

In this cohort study of students who experienced OHCA in school settings in Japan, female sex was associated with lower odds of receiving public-access automated external defibrillator pad application compared with male sex.

**Meaning:**

School staff and students should be informed about the appropriate recognition of OHCA as well as the use of automated external defibrillator via cardiopulmonary resuscitation education.

## Teaser

This cohort study examines sex disparities among youths who experienced cardiac arrest in schools in receipt of public-access automated external defibrillator pad application and cardiopulmonary resuscitation from bystanders in Japan.

## Introduction

Out-of-hospital cardiac arrest (OHCA) is an important public health issue in resource-rich countries,^1,2,3,4^ and the survival rate of OHCA remains low.^5,6,7^ Pediatric patients who experience OHCA accounted for only 3% of all patients who experience OHCA in Japan.^8,9,10^ However, OHCA and sudden OHCA-associated death among children have significant negative impacts on a community in terms of life-years lost, health care costs for survivors, and emotional burden for family members.^11^ Therefore, evaluating factors for improving survival after pediatric OHCA is important in resuscitation science.

As recommended in the cardiopulmonary resuscitation (CPR) guidelines,^1,2,3^ bystanders performing CPR and using a public-access automated external defibrillator (AED) are important factors for improving survival outcomes in OHCAs. Previous studies have reported that women experiencing OHCA in public locations were less likely to receive bystander-initiated CPR compared with men,^12,13,14^ and girls aged 12 to 17 years who experienced OHCA witnessed by nonfamily members were less likely to receive bystander-initiated CPR than boys.^15^ Additionally, El-Assaad et al^16^ reported that children aged 2 to 11 years experiencing OHCAs were less likely to receive AED pad application than children aged 12 to 18 years. Thus, resuscitation behaviors of lay rescuers might change depending on the age and sex of the individual experiencing OHCA. In particular, schoolchildren spend most of their active hours of the day in schools, but to our knowledge, no clinical studies have been conducted to assess sex disparities in receiving public-access AED pad application and bystander-initiated CPR in school settings.

We commenced a nationwide prospective observational study of youths who experience OHCA occurring in school settings in Japan, called the Stop and Prevent Cardiac Arrest, Injury, and Trauma in Schools (SPIRITS) study.^17^ Using this database, we evaluated whether there are sex differences by school level in receiving public-access AED pad application or bystander-initiated CPR among youths experiencing OHCA in school settings.

## Methods

### Ethical Review

This study conformed to the principles of the Declaration of Helsinki,^18^ and the study protocol was approved by the Ethics Committee of Osaka University. Personal identifiers were removed from the database, and the requirement for individual informed consent was waived. This study followed the Strengthening the Reporting of Observational Studies in Epidemiology (STROBE) reporting guideline.

### Study Design

The rationale, design, and profile of the SPIRITS study have been previously described in detail.^17^ Briefly, the SPIRITS study is a nationwide prospective observational study of data from 2 large-scale registries (linked to 1 database): the Injury and Accident Mutual Aid Benefit System of the Japan Sport Council and the All-Japan Utstein Registry of the Fire and Disaster Management Agency. The Injury and Accident Mutual Aid Benefit System of the Japan Sport Council provides benefits (medical expenses, disability compensation, or death compensation) in cases of injury, illness, disease, unintentional injury, or death that occur among students and children under the supervision of schools or nurseries. It covers most students attending schools in Japan (85.9% of nursery school children, 80.7% of kindergarteners, 99.9% of elementary school students, 99.9% of junior high school students, 98.3% of high school students, and 99.4% of technical college students in 2015; an approximate total of 17 million children in Japan).^19^ Data on approximately 1.1 million injury and unintentional injury cases were reported and registered annually from nearly 73 000 schools and nurseries nationwide during the study.^19^ In Japan, 98.0% of high schools, 89.8% of junior high schools, and 72.0% of elementary schools had at least 1 public-access AED in 2008; in 2015, 99.7% of high schools, 99.9% of junior high schools, and 99.9% of elementary schools had at least 1 public-access AED.^20^

The All-Japan Utstein Registry is a population-based OHCA registry based on the international Utstein format^21,22^ and covers the entire population of Japan (approximately 127 million people). In this registry, *cardiac arrest* is defined as the cessation of cardiac mechanical activity confirmed by the absence of signs of circulation, and OHCA data are recorded by emergency medical service (EMS) personnel in cooperation with the physician in charge of the patient. Since prehospital termination of resuscitation by EMS personnel is generally not allowed in Japan, most patients experiencing OHCA cared for by EMS personnel are transported to hospitals, and the data are recorded in this registry, except for patients who are not transported to a hospital by EMS (ie, transported to a hospital by family members or bystanders, non-EMS transporting vehicles, or air ambulance). Thus, the SPIRITS database, which was developed by merging these 2 nationwide registries, has retained the data for most pediatric patients who experienced OHCA in school settings in Japan from April 1, 2008, to December 31, 2015. Data analysis was performed from January 5, 2019, to April 11, 2019.

### Study Participants

Youths from elementary schools (ages 6-12 years), junior high schools (ages 12-15 years), high schools (ages 15-21 years), and technical colleges (ages 15-21 years) in Japan who experienced nontraumatic OHCA from April 1, 2008, to December 31, 2015, were included in this study. Youths in whom a resuscitation attempt was performed by EMS personnel or bystanders were included. Youths whose OHCA occurred after EMS arrival, was caused by trauma (eg, vehicle crashes, falling incidents, and hanging), occurred outside the school campus, or had unknown first documented rhythm were excluded from the analyses.

### Data Collection

We obtained the following data from the SPIRITS database: date and time of emergency call by bystanders, time of contact with patient by EMS personnel, time of arrival at hospital, region, school level, sex, age, whether the OHCA occurred during class time or extracurricular activities, location of cardiac arrest, whether the cardiac arrest was witnessed, cardiac origin of arrest, first documented rhythm, bystander-initiated CPR, and application of public-access AED pads. Based on a 2013 study,^23^ we classified regions in Japan as Hokkaido-Tohoku, Kanto, Tokai-Hokuriku, Kinki, Chugoku-Shikoku, and Kyushu-Okinawa. The end points of this study were bystander interventions for students experiencing OHCA (ie, application of public-access AED pads or initiation of CPR by a bystander).

### Statistical Analysis

Summary statistics were expressed by mean and SD for numerical variables and percentages for categorical variables. Among the eligible students who experienced OHCA, student and EMS characteristics were compared between sexes using the χ^2^ test for categorical variables and *t* test or Mann-Whitney *U* test for numerical variables. In addition, the regional and age distributions between sexes were evaluated by school level. Univariable and multivariable logistic regression analyses were conducted to assess sex differences in the application of public-access AED pads and bystander-initiated CPR. In multivariable analysis, odds ratios (ORs) and their associated 95% CIs were calculated by using multivariable logistic regression, adjusting for region, location of cardiac arrest, school level, whether the cardiac arrest was witnessed, whether the cardiac arrest occurred during class time or extracurricular activities, and cardiac origin of arrest. In addition, since sex differences in the initiation of bystander interventions would vary by school level, ORs and 95% CIs were estimated for each school level by using an interaction of sex and school level in the multivariable model, adjusting for region, location of cardiac arrest, school level, whether the cardiac arrest was witnessed, whether the cardiac arrest occurred during class time or extracurricular activities, and cardiac origin of arrest.

All tests were 2-tailed, and a *P* value of less than .05 was considered statistically significant. Statistical analyses were conducted using Stata statistical software version 15.0 MP (StataCorp).

## Results

The Figure shows the flowchart for the selection of eligible cases of youths who experienced OHCA for the analysis. During the study, a total of 409 pediatric OHCA cases were registered in the SPIRITS database. Among these, 232 cases involving youths (mean [SD] age, 14.5 [2.9] years; 175 [75.4%] male) who experienced OHCA from nontraumatic causes in school campuses were analyzed. There were 42 elementary school students (18.1%), 71 junior high school students (30.6%), and 119 high school or technical school students (51.3%) (Table 1).

**Figure.**
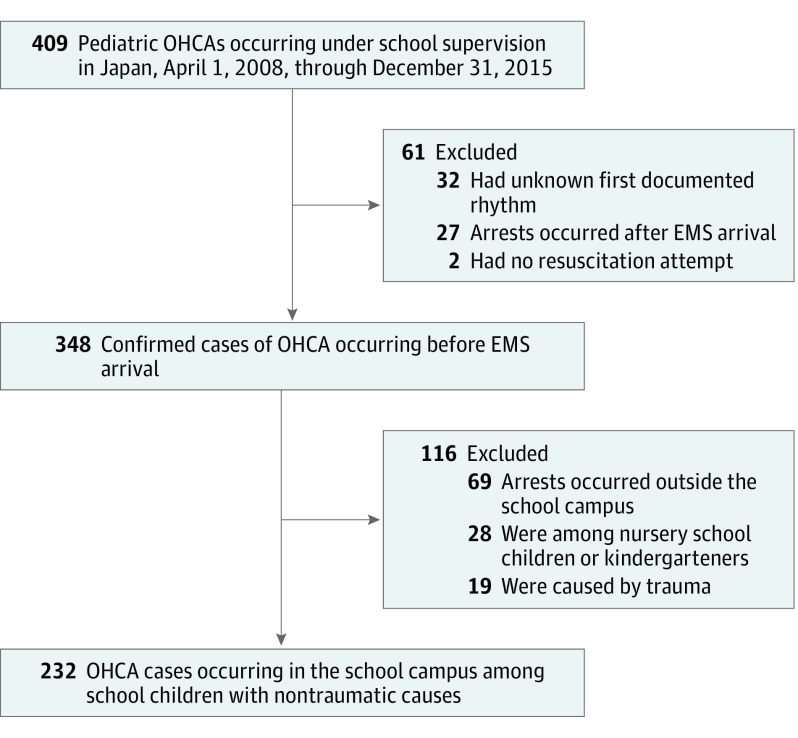
Selection of Eligible Youths Who Experienced Out-of-Hospital Cardiac Arrests (OHCA) From April 1, 2008, to December 31, 2015 EMS indicates emergency medical service.

**Table 1.** Characteristics of Out-of-Hospital Cardiac Arrest in School Settings by Sex

Characteristic	Students, No. (%)			*P* Value
	Total (N = 232)	Female (n = 57)	Male (n = 175)	
Age, mean (SD), y	14.5 (2.9)	13.0 (3.2)	14.9 (2.6)	<.001
School level				
Elementary school	42 (18.1)	22 (38.6)	20 (11.4)	<.001
Junior high school	71 (30.6)	17 (29.8)	54 (30.9)	
High school or technical school	119 (51.3)	18 (31.6)	101 (57.7)	
Region^a^				
Hokkaido-Tohoku	25 (10.9)	8 (14.0)	17 (9.8)	.40
Kanto	75 (32.6)	17 (29.8)	58 (33.5)	
Tokai-Hokuriku	51 (22.2)	9 (15.8)	42 (24.3)	
Kinki	27 (11.7)	10 (17.5)	17 (9.8)	
Chugoku-Shikoku	21 (9.1)	4 (7.0)	17 (9.8)	
Kyushu-Okinawa	31 (13.5)	9 (15.8)	22 (12.7)	
Cardiac arrest occurred during class time	128 (55.2)	38 (66.7)	90 (51.4)	.05
Location of cardiac arrest				
Schoolyard	127 (54.7)	27 (47.4)	100 (57.1)	.19
Gymnasium	44 (19.0)	9 (15.8)	35 (20.0)	
Pool	21 (9.1)	9 (15.8)	12 (6.9)	
Schoolroom	27 (11.6)	9 (15.8)	18 (10.3)	
Other	13 (5.6)	3 (5.3)	10 (5.7)	
Cardiac arrest was witnessed	201 (86.6)	45 (79.0)	156 (89.1)	.05
First documented rhythm				
Ventricular fibrillation	181 (78.0)	38 (66.7)	143 (81.7)	.01
Pulseless electrical activity	22 (9.5)	11 (19.3)	11 (6.3)	
Asystole	29 (12.5)	8 (14.0)	21 (12.0)	
Cardiac origin of arrest	205 (88.4)	49 (86.0)	156 (89.1)	.52
Time from emergency call to EMS personnel contact with the student, mean (SD), min^b^	8.0 (3.2)	8.2 (3.0)	7.9 (3.3)	.53
Time from emergency call to student’s arrival at hospital, mean (SD), min^c^	30.5 (13.2)	29.9 (27.2)	30.7 (14.1)	.67

Abbreviation: EMS, emergency medical service.

^a^ Regional data were missing for 2 individuals.

^b^ Data on time from emergency call to EMS personnel contact with the student were missing for 1 individual.

^c^ Data on time from emergency call to student’s arrival at hospital were missing for 7 individuals.

Table 1 shows student characteristics stratified by sex. The distribution of school level significantly differed by sex, ie, more than half of male students were high school or technical school students (101 of 175 [57.7%]), whereas the plurality of female students were elementary school students (22 of 57 [38.6%]). Overall, OHCAs occurred most frequently in the schoolyard among female students (27 of 57 [47.4%]) and male students (100 of 175 [57.1%]). Out-of-hospital cardiac arrests in female students were less likely to be witnessed (45 [79.0%] vs 156 [89.1%]; *P* = .05) and have ventricular fibrillation rhythm (38 [66.7%] vs 143 [81.7%]; *P* = .01), compared with OHCAs in male students. No statistically significant differences were observed in the distribution of age and region between sexes by school levels (Table 2).

**Table 2.** Characteristics of Students Who Experienced Out-of-Hospital Cardiac Arrest by Sex and School Level

Characteristic	Students, No. (%)			*P* Value
	Total	Female	Male	
**Elementary School**				
Total	42 (100)	22 (52.4)	20 (47.6)	NA
Age, median (range), y	10 (6-12)	10 (6-12)	10 (7-12)	.99
Region^a^				
Hokkaido-Tohoku	8 (20.0)	5 (22.7)	3 (16.7)	.28
Kanto	12 (30.0)	7 (31.8)	5 (27.8)	
Tokai-Hokuriku	9 (22.5)	2 (9.1)	7 (38.9)	
Kinki	3 (7.5)	2 (9.1)	1 (5.6)	
Chugoku-Shikoku	2 (5.0)	1 (4.6)	1 (5.6)	
Kyushu-Okinawa	6 (15.0)	5 (22.7)	1 (5.6)	
**Junior High School**				
Total	71 (100)	17 (23.9)	54 (76.1)	NA
Age, median (range), y	14 (12-17)	14 (12-15)	14 (12-17)	.77
Region				
Hokkaido-Tohoku	8 (11.3)	2 (11.8)	6 (11.1)	.83
Kanto	24 (33.8)	5 (29.4)	19 (35.2)	
Tokai-Hokuriku	15 (21.1)	3 (17.7)	12 (22.2)	
Kinki	7 (9.9)	3 (17.7)	4 (7.4)	
Chugoku-Shikoku	6 (8.5)	2 (11.8)	4 (7.4)	
Kyushu-Okinawa	11 (15.5)	2 (11.8)	9 (16.7)	
**High School or Technical School**				
Total	119 (100)	18 (15.1)	101 (84.9)	NA
Age, median (range), y	17 (15-21)	17 (15-18)	17 (15-21)	.86
Region				
Hokkaido-Tohoku	9 (7.6)	1 (5.6)	8 (7.9)	.62
Kanto	39 (32.8)	5 (27.8)	34 (33.7)	
Tokai-Hokuriku	27 (22.7)	4 (22.2)	23 (22.8)	
Kinki	17 (14.3)	5 (27.8)	12 (11.9)	
Chugoku-Shikoku	13 (10.9)	1 (5.6)	12 (11.9)	
Kyushu-Okinawa	14 (11.8)	2 (11.1)	12 (11.9)	

Abbreviation: NA, not applicable.

^a^ Data on region were missing for 2 individuals.

Table 3 shows differences in the application of public-access AED pads by sex and by school level. In multivariable analysis of students who experienced OHCA, female sex was associated with significantly lower odds of receiving public-access AED pad application compared with male sex (36 [63.2%] vs 141 [80.6%]; adjusted OR, 0.44; 95% CI, 0.20-0.97; *P* = .04). In the subgroup analysis, there were no significant differences in likelihood of receiving public-access AED pad application between sexes among elementary school students (13 girls [59.1%] vs 11 boys [55.0%]; adjusted OR, 0.80; 95% CI, 0.19-3.42; *P* = .77) or junior high school students (13 girls [76.5%] vs 46 boys [85.2%]; adjusted OR, 0.51; 95% CI, 0.12-2.12; *P* = .36). In contrast, among high school and technical school students, female students who experienced OHCA were significantly less likely to receive public-access AED pad application compared with male students (10 female students [55.6%] vs 84 male students [83.2%]; adjusted OR, 0.26; 95% CI, 0.08-0.87; *P* = .03).

**Table 3.** Public-Access Automated External Defibrillator Pad Application for Out-of-Hospital Cardiac Arrest in School Settings by Sex and School Level

Level	Students, No. (%)			Crude		Adjusted	
	Total (N = 232)	Female (n = 57)	Male (n = 175)	OR (95% CI)	*P* Value	OR (95% CI)	*P* Value
Total^a^	177 (76.3)	36 (63.2)	141 (80.6)	0.41 (0.21-0.80)	.008	0.44 (0.20-0.97)	.04
Elementary school^b^	24 (57.1)	13 (59.1)	11 (55.0)	1.18 (0.35-4.02)	.79	0.80 (0.19-3.42)	.77
Junior high school^b^	59 (83.1)	13 (76.5)	46 (85.2)	0.57 (0.15-2.18)	.41	0.51 (0.12-2.12)	.36
High school or technical school^b^	94 (79.0)	10 (55.6)	84 (83.2)	0.25 (0.09-0.73)	.01	0.26 (0.08-0.87)	.03

Abbreviation: OR, odds ratio.

^a^ Adjusted for region, location of cardiac arrest, cardiac origin of arrest, whether the arrest was witnessed, whether the arrest occurred during class time, and school level.

^b^ Adjusted for region, location of cardiac arrest, cardiac origin of arrest, whether the arrest was witnessed, whether the arrest occurred during class time, school level, and an interaction of sex and school level.

Table 4 shows sex differences in the bystander-initiated CPR according to school level. Overall, 48 female (84.2%) and 151 male (86.3%) students experiencing OHCA received bystander-initiated CPR of any type. In multivariable analysis, there were no significant differences between sexes.

**Table 4.** Bystander-Initiated Cardiopulmonary Resuscitation for Out-of-Hospital Cardiac Arrest in School Settings by Sex and School Level

Level	Students, No. (%)			Crude		Adjusted	
	Total (N = 232)	Female (n = 57)	Male (n = 175)	OR (95% CI)	*P* Value	OR (95% CI)	*P* Value
Total^a^	199 (85.8)	48 (84.2)	151 (86.3)	0.85 (0.37-1.95)	.70	0.81 (0.30-2.22)	.68
Elementary school^b^	34 (81.0)	19 (86.4)	15 (75.0)	2.11 (0.43-10.28)	.36	1.50 (0.23-9.78)	.67
Junior high school^b^	63 (88.7)	15 (88.2)	48 (88.9)	0.94 (0.17-5.14)	.94	0.73 (0.11-4.80)	.74
High school or technical school^b^	102 (85.7)	14 (77.8)	88 (87.1)	0.52 (0.15-1.81)	.30	0.56 (0.12-2.50)	.45

Abbreviation: OR, odds ratio.

^a^ Adjusted for region, location of cardiac arrest, cardiac origin of arrest, whether the arrest was witnessed, whether the arrest occurred during class time, and school level.

^b^ Adjusted for region, location of cardiac arrest, cardiac origin of arrest, whether the arrest was witnessed, whether the arrest occurred during class time, school level, and an interaction of sex and school level.

## Discussion

From the nationwide SPIRITS database in Japan, we assessed differences in receiving public-access AED pad application and bystander-initiated CPR between male and female students who experienced an OHCA in a school setting. We found that female sex was associated with lower odds of receiving public-access AED pad application compared with male sex, and the association remained after adjusting for potential confounders. There were no significant differences in receiving bystander-initiated CPR between sexes. To our knowledge, this was the first attempt to assess sex differences in receiving public-access AED pad application and bystander-initiated CPR in schools, where school-aged youths spend most of their active hours of the day. The results of this study would be useful for future revisions of school CPR education programs and for improving survival from pediatric OHCA in school settings.

To our knowledge, there are few previous studies that have evaluated AED pad application among youths who experienced OHCA. In the only previous study assessing factors associated with AED pad application for pediatric OHCA, to our knowledge, El-Assaad et al^16^ reported that a factor associated with AED pad application in the United States was age: children aged 2 to 11 years who experienced OHCA were less likely to receive AED pad application compared with youths aged 12 to 18 years. El-Assaad et al^16^ speculated that this was because there was a lack of CPR knowledge and confusion about AED use for children among bystanders. There were no significant differences in receiving AED application between sexes, and they did not assess sex differences in receiving AED pad application by school level or age group.

Our study found that female sex among youths who experienced OHCA in schools was associated with significantly lower odds of receiving public-access AED pad application than male sex among youths who experienced OHCA in schools. The reason for this sex difference in receiving public-access AED pad application is unclear. We speculate that it may be because bystanders must undress a person experiencing OHCA to apply AED pads, but chest compressions can be conducted without undressing. Bystanders might be embarrassed and afraid of applying AED pads on school-aged female youths because undressing women and girls is an unfavorable behavior in public places, even if they are experiencing cardiac arrest, and for male bystanders to do so, it might be misunderstood as sexual assault.

In this study, more than 80% of youths who experienced OHCA received CPR from a bystander, and there were no differences in receiving CPR between sexes, regardless of school level. A 2019 study in Japan^15^ reported that girls were less likely to receive bystander-initiated CPR than boys among youths aged 12 to 17 years experiencing OHCA witnessed by nonfamily members, and the authors speculated that bystanders might be hesitant to perform CPR on a stranger because of the possibility of being sued, risk of infectious disease, and risk of hurting the person if CPR was unnecessary or performed incorrectly.^24^ However, since almost all staff in schools in Japan have received comprehensive training in basic life support,^25^ most youths experiencing OHCA should receive bystander-initiated CPR regardless of sex. However, approximately 15% of youths who experienced OHCA in schools did not receive bystander-initiated CPR. Therefore, to ensure everyone who experiences OHCA receives bystander-initiated CPR, systematic CPR education and training should be continuously carried out in schools for staff and students to achieve the Japanese Circulation Society’s goal of no deaths of sudden cardiac arrests in schools.^26^

Because female sex among students who experienced OHCA in schools was associated with lower odds of receiving public-access AED pad application than male sex, we must educate school staff and students on the recognition of OHCA as well as the proper use of AED via CPR education and training so that they can apply AED pads for female students experiencing OHCA without hesitation. Almost all elementary, junior high, and high schools in Japan have installed at least 1 AED.^25^ To further increase the implementation of AED pad application regardless of sex, public-access AEDs should be located in school buildings as well as in school athletic areas, such as the schoolyard, pool, and gymnaseum,^27^ so that AED interventions can be delivered within 5 minutes, based on Japanese recommendations on the deployment criteria of public-access AEDs.^28^

### Limitations

This study has several inherent limitations. First, our study did not obtain information regarding the sex of bystanders performing CPR or their experience with previous CPR training. Second, the concern of undressing an older girl might be a barrier to AED placement. For example, there are some all-girls schools in Japan, but we did not obtain information on whether schools where the cardiac arrests occurred were single-gender schools. However, only 7% of schools in Japan are all-girls schools,^29^ so the impact of all-girls schools on our data would be small. Third, the implementation of public-access AED pad application and bystander-initiated CPR were our main outcome, and the survival outcome after OHCA between sexes was not evaluated in this study. Survival after OHCA is significantly affected by other factors, such as witness status, first documented rhythm, and treatments by EMS personnel and medical institutions as well as the provision of bystander-initiated CPR. Fourth, our findings may not be fully generalizable to other health care settings, given the differences in patient characteristics and medical care systems. Fifth, there is a possibility of input errors in the items for data linkage for the development of the SPIRITS database, which could lead to underestimation of OHCA cases to a certain degree. Moreover, the exclusion of participants who were not transported to the hospital by EMS personnel could also cause underestimation of OHCA incidence. Additionally, there may be unmeasured confounding factors that might have influenced the association of sex with AED pad application.

## Conclusions

Female sex among students who experienced OHCA in schools in Japan was associated with lower odds of receiving public-access AED pad application compared with male sex, and the association remained after adjusting for potential confounders. Observational studies in other countries are essential to verify sex disparities in receiving public-access AED pad application among school-aged youths who experience OHCA and to confirm our results.

## References

[1] Neumar RW, Shuster M, Callaway CW, Part 1: executive summary: 2015 American Heart Association Guidelines update for cardiopulmonary resuscitation and emergency cardiovascular care. Circulation. 2015;132(18)(suppl 2):-. doi:10.1161/CIR.0000000000000252 26472989

[2] Hazinski MF, Nolan JP, Aickin R, Part 1: executive summary: 2015 international consensus on cardiopulmonary resuscitation and emergency cardiovascular care science with treatment recommendations. Circulation. 2015;132(16)(suppl 1):S2-S39. doi:10.1161/CIR.0000000000000270 26472854

[3] Monsieurs KG, Nolan JP, Bossaert LL, ; ERC Guidelines 2015 Writing Group European Resuscitation Council Guidelines for Resuscitation 2015: section 1: executive summary. Resuscitation. 2015;95:1-80. doi:10.1016/j.resuscitation.2015.07.038 26477410

[4] Japan Resuscitation Council Japanese Guidelines for Emergency Care and Cardiopulmonary Resuscitation [in Japanese]. Tokyo, Japan: Igakusyoin; 2016.

[5] Chan PS, McNally B, Tang F, Kellermann A; CARES Surveillance Group Recent trends in survival from out-of-hospital cardiac arrest in the United States. Circulation. 2014;130(21):1876-1882. doi:10.1161/CIRCULATIONAHA.114.009711 25399396 PMC4276415

[6] Jayaram N, McNally B, Tang F, Chan PS Survival after out-of-hospital cardiac arrest in children. J Am Heart Assoc. 2015;4(10):e002122. doi:10.1161/JAHA.115.002122 26450118 PMC4845116

[7] Perkins GD, Lockey AS, de Belder MA, Moore F, Weissberg P, Gray H; Community Resuscitation Group National initiatives to improve outcomes from out-of-hospital cardiac arrest in England. Emerg Med J. 2016;33(7):448-451. doi:10.1136/emermed-2015-204847 26400865 PMC4941191

[8] Fire and Disaster Management Agency of Japan Effect of first aid for cardiopulmonary arrest [in Japanese]. https://www.fdma.go.jp/publication/rescue/items/kkkg_h30_01_kyukyu.pdf. Accessed January 26, 2019.

[9] Nitta M, Iwami T, Kitamura T, ; Utstein Osaka Project Age-specific differences in outcomes after out-of-hospital cardiac arrests. Pediatrics. 2011;128(4):e812-e820. doi:10.1542/peds.2010-3886 21890823

[10] Herlitz J, Svensson L, Engdahl J, Characteristics of cardiac arrest and resuscitation by age group: an analysis from the Swedish Cardiac Arrest Registry. Am J Emerg Med. 2007;25(9):1025-1031. doi:10.1016/j.ajem.2007.03.008 18022497

[11] Atkins DL, Berger S Improving outcomes from out-of-hospital cardiac arrest in young children and adolescents. Pediatr Cardiol. 2012;33(3):474-483. doi:10.1007/s00246-011-0084-8 21842254

[12] Blewer AL, McGovern SK, Schmicker RH, ; Resuscitation Outcomes Consortium Investigators Gender disparities among adult recipients of bystander cardiopulmonary resuscitation in the public. Circ Cardiovasc Qual Outcomes. 2018;11(8):e004710. doi:10.1161/CIRCOUTCOMES.118.004710 30354377 PMC6209113

[13] Wissenberg M, Hansen CM, Folke F, Survival after out-of-hospital cardiac arrest in relation to sex: a nationwide registry-based study. Resuscitation. 2014;85(9):1212-1218. doi:10.1016/j.resuscitation.2014.06.008 24960430

[14] Safdar B, Stolz U, Stiell IG, Differential survival for men and women from out-of-hospital cardiac arrest varies by age: results from the OPALS study. Acad Emerg Med. 2014;21(12):1503-1511. doi:10.1111/acem.12540 25491713

[15] Okubo M, Matsuyama T, Gibo K, Sex differences in receiving layperson cardiopulmonary resuscitation in pediatric out-of-hospital cardiac arrest: a nationwide cohort study in Japan. J Am Heart Assoc. 2019;8(1):e010324. doi:10.1161/JAHA.118.010324 30587069 PMC6405730

[16] El-Assaad I, Al-Kindi SG, McNally B, ; CARES Surveillance Group Automated external defibrillator application before EMS arrival in pediatric cardiac arrests. Pediatrics. 2018;142(4):e20171903. doi:10.1542/peds.2017-1903 30262669

[17] Kiyohara K, Sado J, Kitamura T, ; SPIRITS Investigators Epidemiology of pediatric out-of-hospital cardiac arrest at school: an investigation of a nationwide registry in Japan. Circ J. 2018;82(4):1026-1032. doi:10.1253/circj.CJ-17-1237 29445066

[18] World Medical Association World Medical Association Declaration of Helsinki: ethical principles for medical research involving human subjects. JAMA. 2013;310(20):2191-2194. doi:10.1001/jama.2013.281053. 24141714

[19] Japan Sport Council School Safety Department. https://www.jpnsport.go.jp/corp/english/activities/tabid/395/Default.aspx. Accessed January 26, 2019.

[20] The Ministry of Education, Culture, Sports, Science and Technology-Japan Survey on the public-access AED dissemination in schools [in Japanese]. http://www.mext.go.jp/a_menu/kenko/anzen/1339095.htm. Accessed March 30, 2019.

[21] Perkins GD, Jacobs IG, Nadkarni VM, ; Utstein Collaborators Cardiac arrest and cardiopulmonary resuscitation outcome reports: update of the Utstein Resuscitation Registry Templates for Out-of-Hospital Cardiac Arrest: a statement for healthcare professionals from a task force of the International Liaison Committee on Resuscitation (American Heart Association, European Resuscitation Council, Australian and New Zealand Council on Resuscitation, Heart and Stroke Foundation of Canada, InterAmerican Heart Foundation, Resuscitation Council of Southern Africa, Resuscitation Council of Asia); and the American Heart Association Emergency Cardiovascular Care Committee and the Council on Cardiopulmonary, Critical Care, Perioperative and Resuscitation. Circulation. 2015;132(13):1286-1300. doi:10.1161/CIR.0000000000000144 25391522

[22] Jacobs I, Nadkarni V, Bahr J, ; International Liaison Committee on Resuscitation; American Heart Association; European Resuscitation Council; Australian Resuscitation Council; New Zealand Resuscitation Council; Heart and Stroke Foundation of Canada; InterAmerican Heart Foundation; Resuscitation Councils of Southern Africa; ILCOR Task Force on Cardiac Arrest and Cardiopulmonary Resuscitation Outcomes Cardiac arrest and cardiopulmonary resuscitation outcome reports: update and simplification of the Utstein templates for resuscitation registries: a statement for healthcare professionals from a task force of the International Liaison Committee on Resuscitation (American Heart Association, European Resuscitation Council, Australian Resuscitation Council, New Zealand Resuscitation Council, Heart and Stroke Foundation of Canada, InterAmerican Heart Foundation, Resuscitation Councils of Southern Africa). Circulation. 2004;110(21):3385-3397. doi:10.1161/01.CIR.0000147236.85306.15 15557386

[23] Okamoto Y, Iwami T, Kitamura T, Regional variation in survival following pediatric out-of-hospital cardiac arrest. Circ J. 2013;77(10):2596-2603. doi:10.1253/circj.CJ-12-1604 23823852

[24] Taniguchi T, Sato K, Fujita T, Okajima M, Takamura M Attitudes to bystander cardiopulmonary resuscitation in Japan in 2010. Circ J. 2012;76(5):1130-1135. doi:10.1253/circj.CJ-11-0054 22382380

[25] The Ministry of Education, Culture, Sports, Science and Technology-Japan Elementary and Secondary Education Planning Division [in Japanese]. http://www.mext.go.jp/component/a_menu/education/detail/__icsFiles/afieldfile/2017/03/24/1289307_12.pdf. Accessed January 26, 2019.

[26] Mitamura H, Iwami T, Mitani Y, Takeda S, Takatsuki S Aiming for zero deaths: prevention of sudden cardiac death in schools: statement from the AED Committee of the Japanese Circulation Society. Circ J. 2015;79(7):1398-1401. doi:10.1253/circj.CJ-15-0453 26084338

[27] Hazinski MF, Markenson D, Neish S, ; American Heart Association; American Academy of Pediatrics; American College of Emergency Physicians; American National Red Cross; National Association of School Nurses; National Association of State EMS Directors; National Association of EMS Physicians; National Association of Emergency Medical Technicians; Program for School Preparedness and Planning, National Center for Disaster Preparedness, Columbia University Mailman School of Public Health Response to cardiac arrest and selected life-threatening medical emergencies: the medical emergency response plan for schools: a statement for healthcare providers, policymakers, school administrators, and community leaders. Ann Emerg Med. 2004;43(1):83-99. doi:10.1016/j.annemergmed.2003.11.001 14707947

[28] Japanese Circulation Society Recommendations on deployment criteria of public-access AEDs [in Japanese]. https://www.jhf.or.jp/check/aed/arrangement/. Accessed January 26, 2019.

[29] The Ministry of Education, Culture, Sports, Science and Technology-Japan Report on school basic survey [in Japanese]. https://www.e-stat.go.jp/stat-search/files?tstat=000001011528. Accessed March 30, 2019.

